# Supplementary material for: Photolysis by UVA–Visible Light of TNT in Ethanolic, Aqueous-Ethanolic, and Aqueous Solutions According to Electrospray and Aerodynamic Thermal Breakup Droplet Ionization Mass Spectrometry
Source: Molecules. 2022 Nov 17;27(22):7992. doi: 10.3390/molecules27227992 (PMC9697284; doi:10.3390/molecules27227992)
Supplement: Supplementary file 1 [file molecules-27-07992-s001.zip › molecules-2037396-supplementary.pdf]

# Photolysis by UVA-Visible Light of TNT in Ethanolic, Aqueous-Ethanolic, and Aqueous Solutions According to Electrospray and Aerodynamic Thermal Breakup Droplet Ionization Mass Spectrometry

Dmitriy G. Sheven * and Viktor V. Pervukhin

Nikolaev Institute of Inorganic Chemistry SB RAS, Acad. Lavrentieva Ave. 3, 630090 Novosibirsk, Russia; pervv@niic.nsc.ru

* Correspondence: sh_dim@ngs.ru

**Text S1**

The ATBDI method gist is based on two well-known formulas:

The Young–Laplace equation, (Rusanov, A.I., 1967. Phase Equilibria and Surface Phenomena. Khimiya, Leningrad [in Russian]):

$$P_d = P_0 + \frac{2\sigma}{r} \quad (1s)$$

where $P_d$ is pressure inside a droplet of radius $r$, $P_0$ is ambient pressure, and $\sigma$ is surface tension of the liquid;

and the van der Waals equation (Straub, J., Rosner, N., Grigull, U., 1980. Oberflächenspannung von leichtem und schwerem Wasser, Wärme- und Stoffübertragung. 13, 241-252):

$$\sigma \approx (T_c - T)^{3/2} \quad (2s)$$

where $T_C$ is critical temperature.

The pressure inside a micron-sized drop can reach several atmospheres (eq. 1s), and it is controlled by surface tension ($\sigma$). As $\sigma$ decreases (it heats up to a temperature close to $T_C$, eq. 2s), this pressure leads to the explosive breakup of the drop. According to the Dodd interpretation (Dodd E.E. The statistics of liquid spray and dust electrification by the Hopper and Laby method, J. Appl. Phys. 24 (1953) 73–80), the small droplets obtained are statistically charged (if there are charged particles in the initial solution).

As presented in Fig. S1, in ATBDI, the solution is broken down into droplets twice: first in the Collison nebulizer (Fig. S1A2, the spray nozzle system) and then in the thermal droplet breakup system (Fig. S1B). At the first stage, the mechanical spraying produces droplets with a diameter of ~3 μm. Further fragmentation of the droplet at the second stage is due to a decrease in the surface tension of the liquid with a temperature increase (J. Kalová, R. Mareš, The Temperature Dependence of the Surface Tension of Water, AIP Conference Proceedings 2047, 020007 (2018). https://doi.org/10.1063/1.5081640). The droplet size and hence the ion current at this stage depends on the conditions in the suction tube/ATBDI reactor (Fig. S1B4), in particular on its temperature ($T_{suction}$), as vividly illustrated in Fig. S5. It shows the behavior of the ion current of deprotonated TNT, $(M – H)^–$ as a function of $T_{suction}$ of the copper tube. At $T_{suction}$ < 50 °C, the ion current is relatively small because it is caused only by the spraying of the solution in the Collison nebulizer. The increase in $T_{suction}$ initiates the thermal breakup of the droplets and enhances the ion current by an order of magnitude.

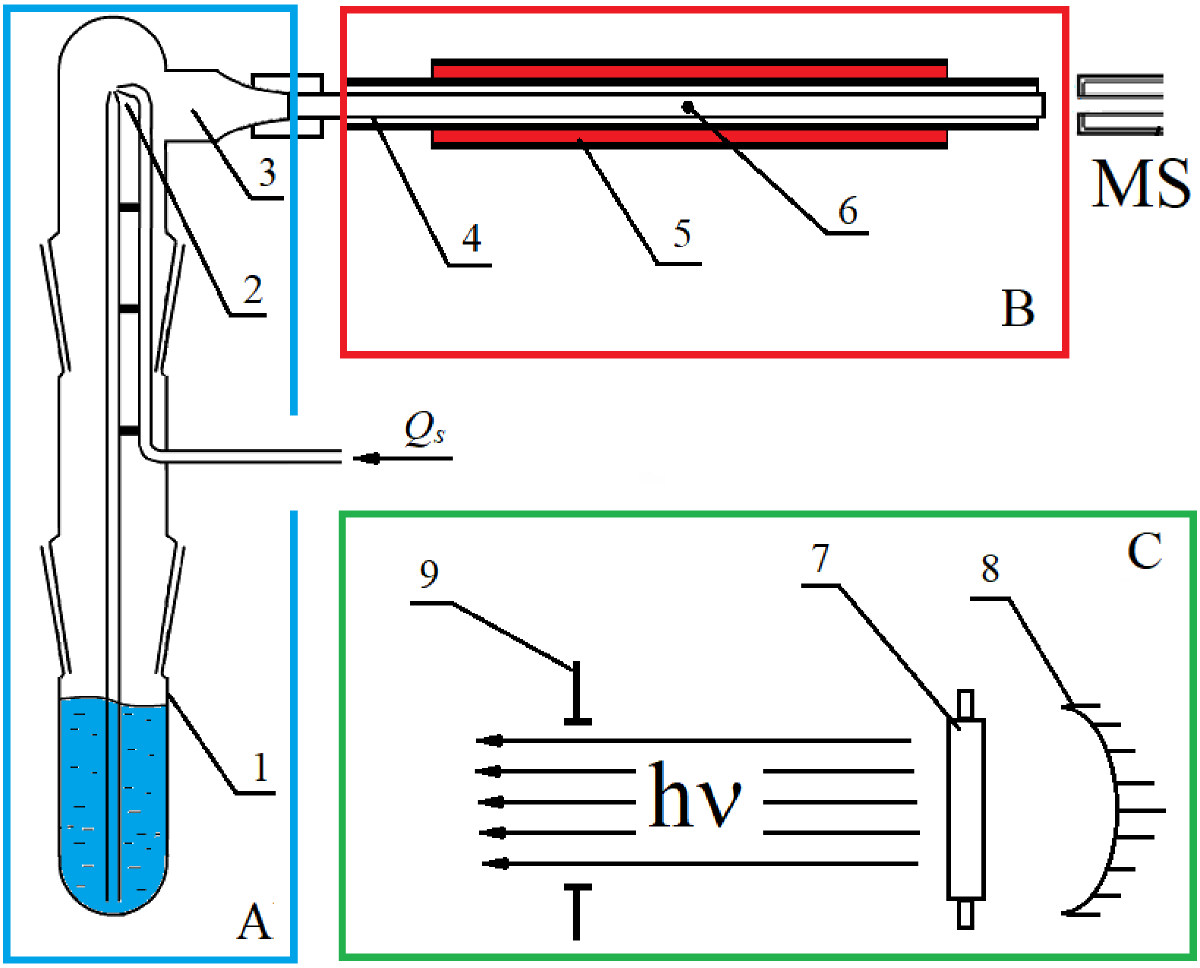


**Figure S1.** A schematic diagram of the setup for investigating photochemical reactions using ATBDI. A: The Collison nebulizer (1: a reservoir for the photolyzed liquid, a molybdenum glass flask; 2: a system of spray nozzles with an inner diameter of 0.3 mm; 3: a nebulizer outlet). B: A thermal droplet degradation system/thermal reactor (4: suction tubes/the ATBDI reactor, copper or quartz tubes can be used (outer diameter: 3 mm, inner diameter: 2 mm, and length: 130 mm for both); 5: a heater; 6: a temperature measurement point). C: Illuminator OI-18A (7: mercury-quartz lamp DRK-120; 8: a spherical reflector; 9: a diaphragm). $Q_s$ is the spray gas flow, and MS is the inlet capillary of the mass spectrometer.

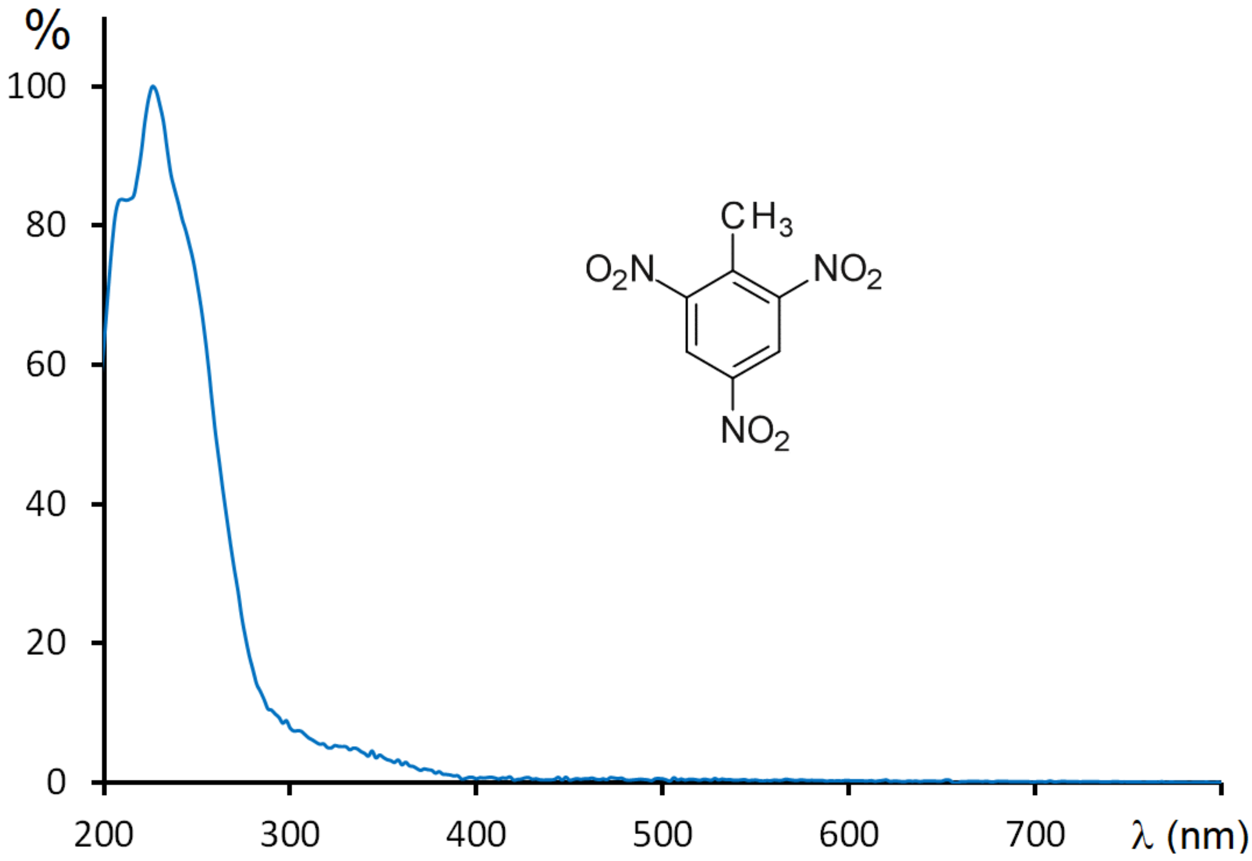


**Figure S2.** TNT absorption spectrum in the UV-vis region. TNT ($10^2$ mg $L^{-1}$) dissolved in water/ethanol (1:1, v/v). Photolysis was not done.

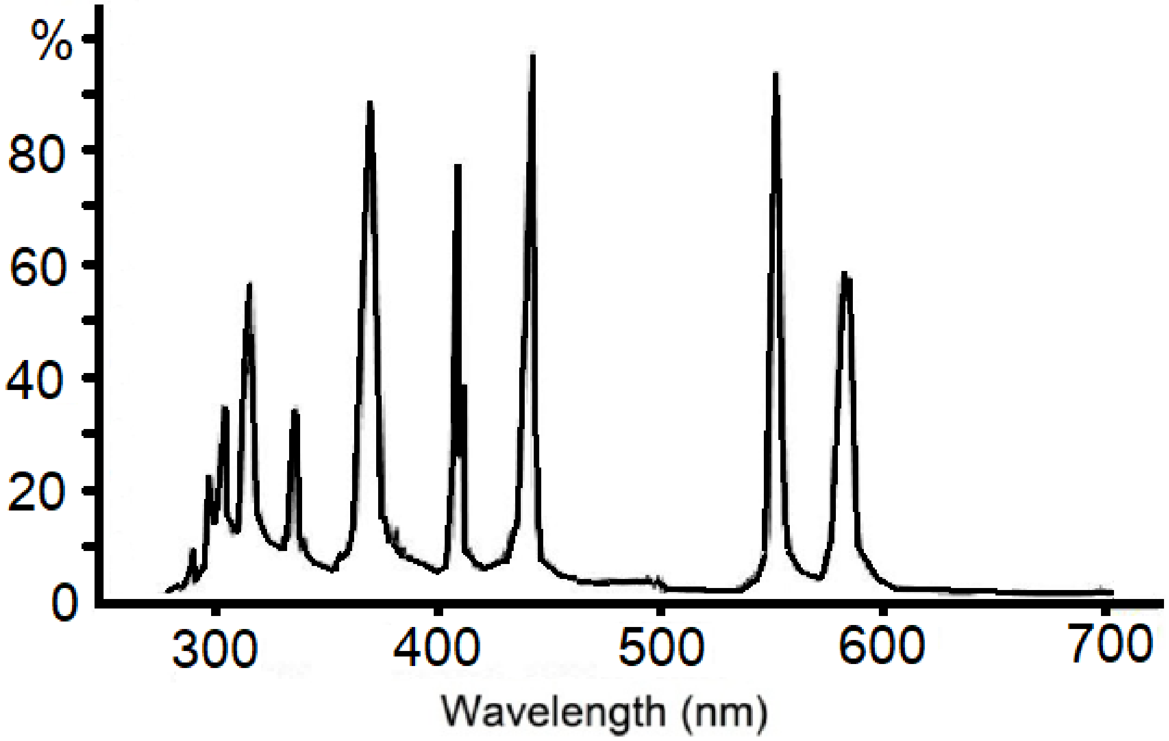


**Figure S3.** The emission spectrum of a mercury lamp DRK-120 (an arc mercury-quartz lamp) in the UV-vis region.

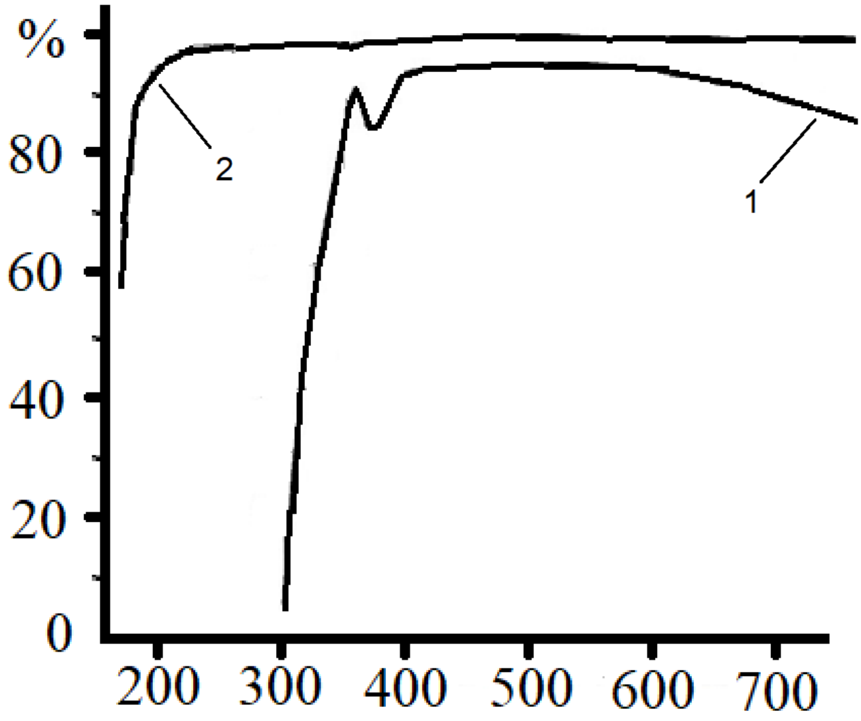


**Figure S4.** Transmission spectrum of (1) molybdenum and KU-1 quartz glass (2) in the UV-vis region.

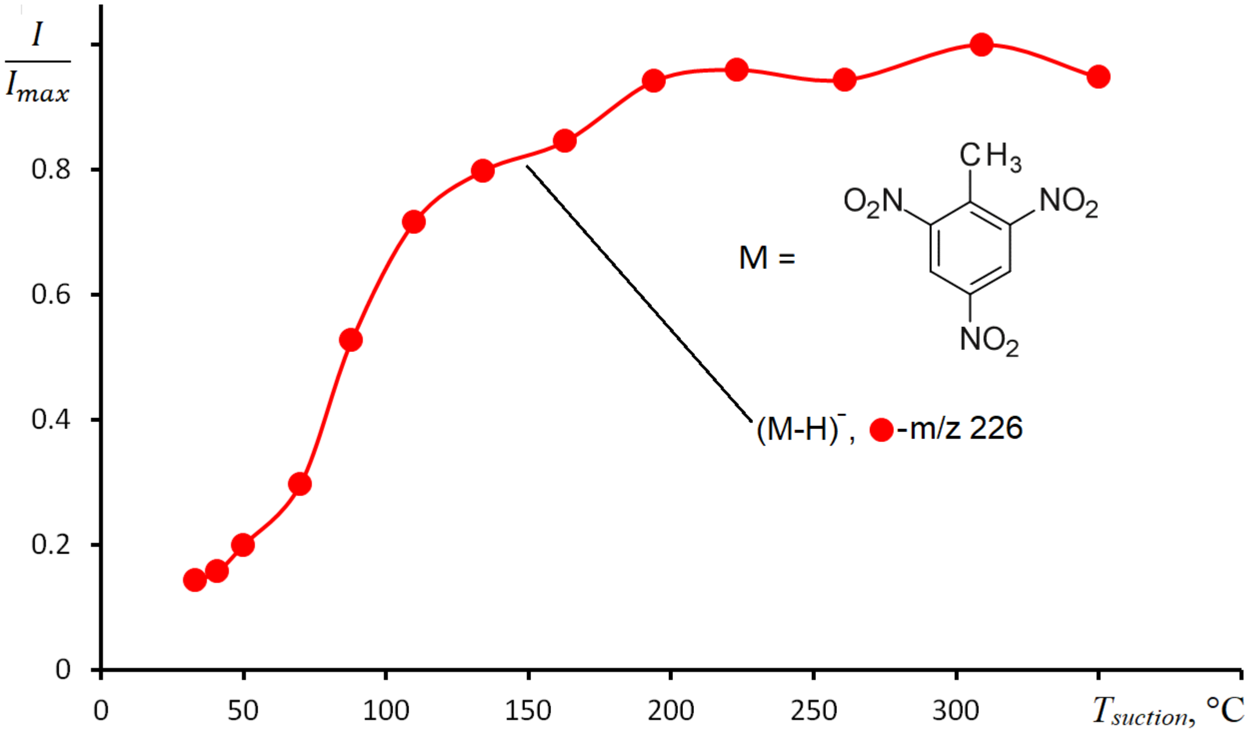


**Figure S5.** Ionization efficiency of ATBDI toward TNT. The graph depicts the ionic current of deprotonated TNT, $(M - H)^{-}$, depending on the temperature of the copper suction tube, $T_{suction}$. The solution used: TNT in water, concentration 10 mg $L^{-1}$. The ion current was normalized to the maximum seen in the graph.

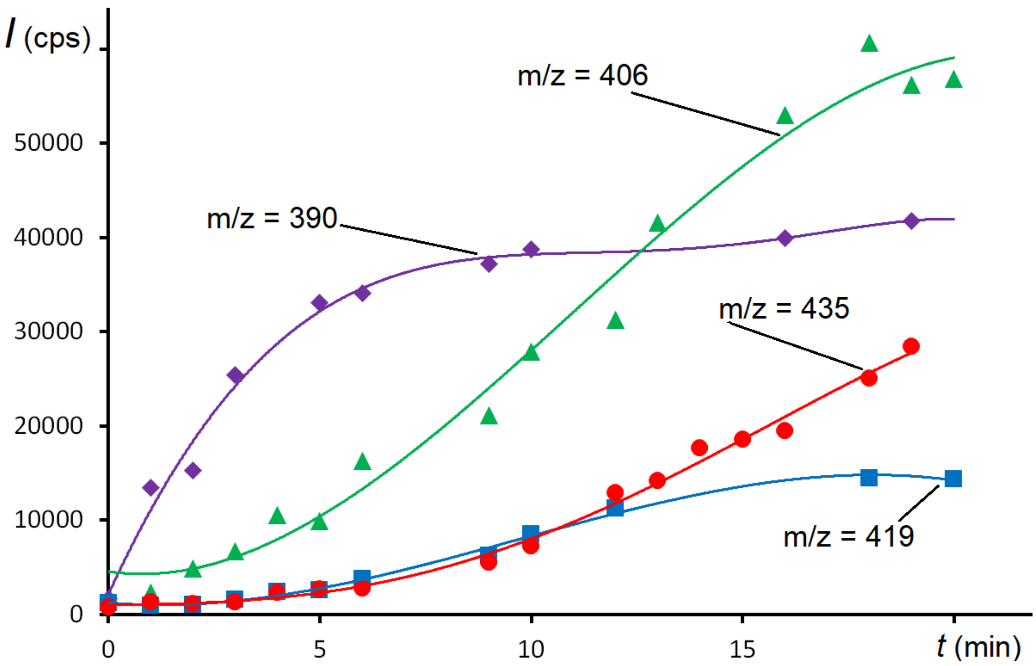


**Figure S6.** Time changes of the ESI mass spectrometry peaks of a UVA-visible photolyzed solution of TNT in ethanol ($10^2$ mg $L^{-1}$). The structures of the compounds corresponding to the m/z values shown are indicated in the text (similar figures can be obtained for an aqueous solution of TNT and a water-ethanol solution).

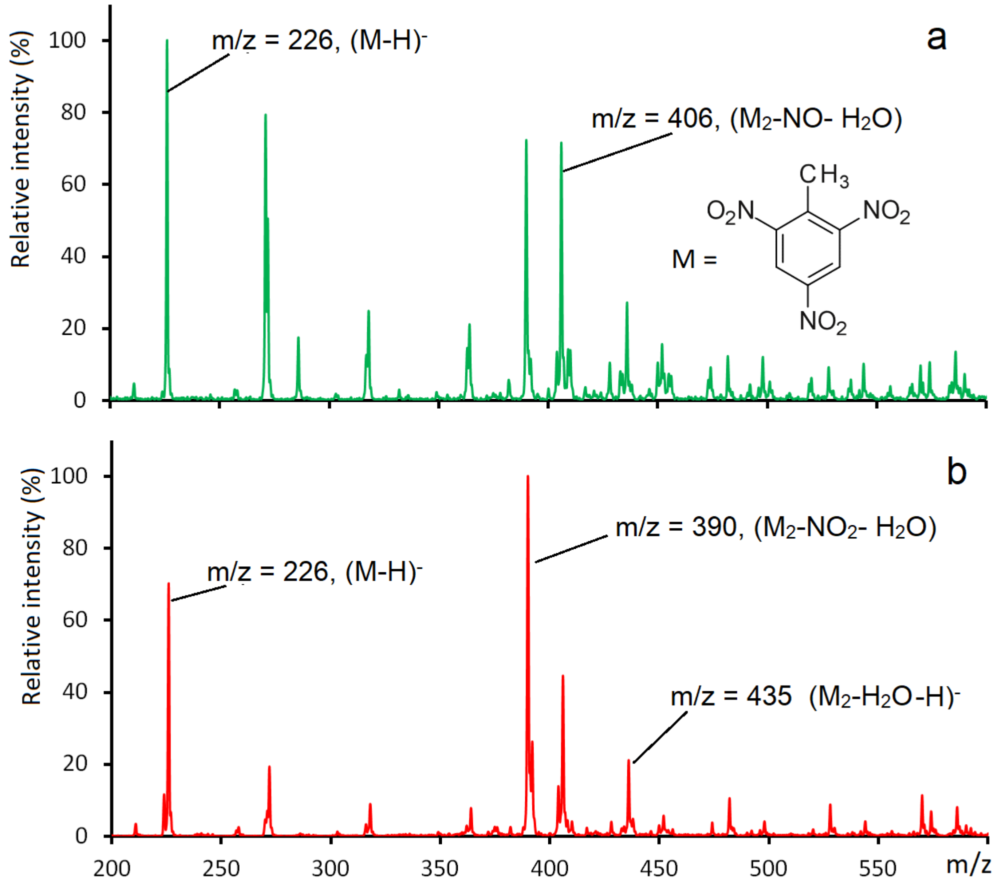


**Figure S7.** ATBDI mass spectra for TNT in ethanol ($10^2$ mg $L^{-1}$). The temperature of suction tube ($T_{suction}$): 88 °C (a); 223°C (b). The fragmentor voltage is 100 V. The suction tube is made of quartz.

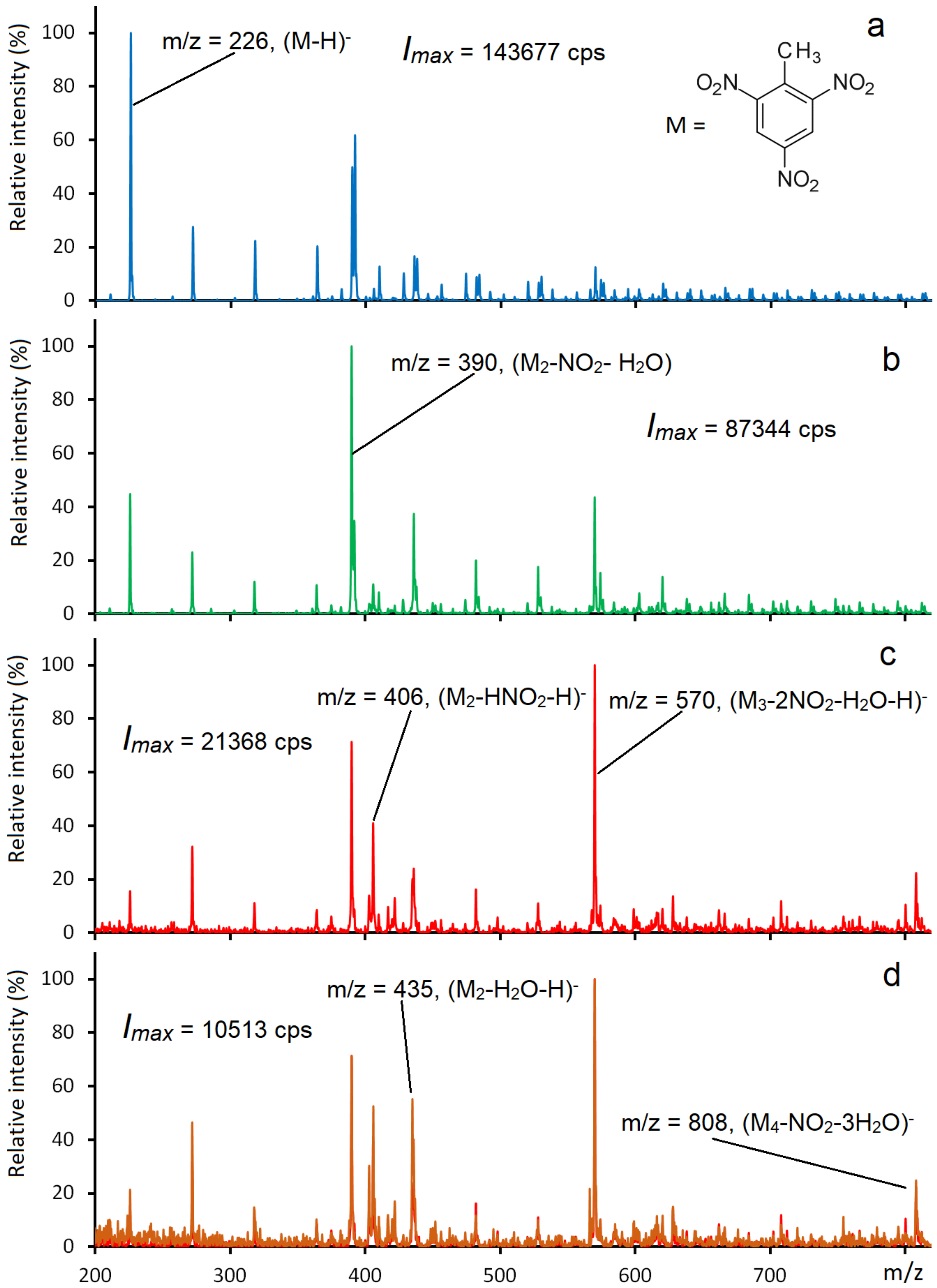


**Figure S8.** Change in the mass spectra of ATBDI for TNT in ethanol ($10^2$ mg $L^{-1}$) during photolysis through quartz. Photolysis time: 2.5min (a); 5.5 min (b); 14.5min (c); 19.5 min (d). The temperature of suction tube ($T_{suction}$): 242 °C. The fragmentor voltage is 100 V. The suction tube is made of quartz.
